# An Account of the Successful Application of Electricity in a Case of Wry Neck

**Published:** 1790

**Authors:** William Gilby

**Affiliations:** Physician to the General Hospital at Birmingham


### XII. *An Account of the successful Application of Electricity in a Case of wry Neck. By* William Gilby, *M. D. Physician to the General Hospital at Birmingham.*

MRS. M. G., aged twenty-eight years, in every other respect healthy, had laboured under the disease termed wry neck about three months before the time of my seeing her, which was early in March, 1784. The contraction seemed then to be chiefly in the left sterno-cleido-mastoideus muscle, but it was afterwards very apparent in the trapezius and subjacent muscles on the same side. The contraction was so great as to confine the head irresistibly upon the shoulder; and any attempt to bring it into its proper place, by the hand, was attended with great pain and sense of stiffness in the affected muscles.

The patient laboured under this disease near nine months, and above seven of this time was in a state of pregnancy. As soon as she was capable of going abroad after her delivery I resolved (as various other remedies had proved ineffectual) upon trying electricity, which might, without the smallest danger, have been made uſe of, in the manner that it proved ef-fectual, at a more early period.

I began by drawing ſparks from the left ſterno-cleido-maſtoideus muſcle; but ſeeing that this experiment had the effect of making it contract ſtill the more, I thought it adviſable to apply the metallic ſubſtance to the muſcle of the ſame name on the oppoſite ſide. In leſs than five minutes I plainly diſcovered at every approximation of the metal ſeveral efforts in the fibres to contract. Sparks were drawn from this and the antagoniſts to the other contracted muſcles until the ſkin preſented an appearance ſimilar to that of the nettle raſh. The patient, after the ſecond or third time, ſaid ſhe found herſelf capable of making ſome little reſiſtance to the action of the muſcles which confined the head upon the ſhoulder. This, with the ſub-ſequent ſigns of amendment, induced me to re-peat the operation night and morning until the cure was perfected. The ſkin becoming more and more accuſtomed to the ſtimulus, I was enabled to draw the ſparks ſomewhat longer each time until, at length, I went on with little or no intermiſſion for the ſpace of half an hour. In the beginning the ſoreneſs became very great at the end of five minutes. The relaxed muſ-

cles cles gradually regained their original tone or contractile power; and at length being slightly assisted in their action by means of a broad ligature passed round the head, which the patient could draw with the hand or fasten under the right arm at pleasure, she was not only enabled to replace the head in its proper position, but to keep it there.

In about a month after the first time of using electricity she had so far the command over the muscles on each side as to be capable of moving the head in every direction, although not without some little pain and inconvenience, which by degrees went off entirely. Some years have now elapsed, and there has been no return of the complaint.

I have since met with a similar case where the contraction was not so great, but of longer duration, which was treated in the same manner, and with the same success.

It may be necessary to observe, that, in cases of this sort, the patient should facilitate the action of the relaxed muscles by keeping the head some hours in the day upon a pillow filled with bran or saw-dust. The head ought to be so placed upon the pillow as to occasion a constant resistance to the contraction. Great attention ought to be paid to this matter during the night.

I have feen cafes where there has been a want of proper motion in the knee, arifing from contractions of the flexor mufcles, which have been fuccefsfully treated by drawing electrical fparks for the fpace of half an hour every day from the extenfors. Thefe contractions generally arife from long confinement of the limb in the fame pofition, as in cafes of accidents.

In thefe cafes the electrical fparks muft be very ftrong to produce any good effect; the apparatus, therefore, ought to be large and kept perfectly dry.

